# Supplementary material for: Differential Expression of Two Copies of the irmA Gene in the Enteroaggregative E. coli Strain 042
Source: Microbiol Spectr. 2022 Jun 29;10(4):e00454-22. doi: 10.1128/spectrum.00454-22 (PMC9431211; doi:10.1128/spectrum.00454-22)
Supplement: Supplemental file 1 — Supplemental material. Download spectrum.00454-22-s0001.pdf, PDF file, 1.6 MB [file spectrum.00454-22-s0001.pdf]

**Supplementary Information.**

Differential expression of two copies of the *irmA* gene in the enteroaggregative *E. coli* strain 042.

Bernabeu, M.[1], Aznar, S.[2], Prieto, A.[1], Hüttener, M.[1]* and Juárez, A.[1,2]*

[1]Department of Genetics, Microbiology and Statistics. Universitat de Barcelona. Barcelona, Spain.

[2]Institute for Bioengineering of Catalonia, The Barcelona Institute of Science and Technology, Barcelona, Spain.

*Corresponding authors: Professor Antonio Juárez (ajuarez@ub.edu) and Dr. Mário Hüttener (mhuttener@me.com).

| Bacterial strain | Description | Source/Reference |
|---|---|---|
| DH5α | *fhuA2 lac(del)U169 phoA glnV44 Φ80' lacZ(del)M15 gyrA96 recA1 relA1 endA1 thi-1 hsdR17* | (1) |
| BL21 (DE3) | *hsdS gal (λcIts857 ind1 Sam7 nin5 lac-UV5-T7)* | (2) |
| EAEC 042 | *E. coli* EAEC, $Cm^r$ $Sm^r$ $Tc^r$ | Prof. I. Henderson |
| EAEC 042 *irmA_2244*xFLAG | 042 with epitope FLAG added to *irmA_2244* C-terminal | This work |
| EAEC 042 *irmA_2244*xFLAG *hha* | 042 *irmA_2244*xFLAG carrying *hha* deletion | This work |
| EAEC 042 *irmA_2244*xFLAG *hha hha2* | 042 *irmA_2244*xFLAG carrying *hha* and *hha2* deletion | This work |
| EAEC 042 *irmA_2244*xFLAG *hns* | 042 *irmA_2244*xFLAG carrying *hns* deletion | This work |
| EAEC 042 *irmA_2244*xFLAG *oxyR* | 042 *irmA_2244*xFLAG carrying *oxyR* deletion | This work |
| EAEC 042 *irmA_2244*xFLAG *irmA_4509* | 042 *irmA_2244*xFLAG carrying *irmA_4509* deletion | This work |
| EAEC 042 *irmA_4509*xFLAG | 042 with epitope FLAG added to *irmA_4509* C-terminal | This work |
| EAEC 042 *irmA_4509*xFLAG *hha* | 042 *irmA_4509*xFLAG carrying *hha* deletion | This work |
| EAEC 042 *irmA_4509*xFLAG *hha hha2* | 042 *irmA_4509*xFLAG carrying *hha* and *hha2* deletion | This work |
| EAEC 042 *irmA_4509*xFLAG *hns* | 042 *irmA_4509*xFLAG carrying *hns* deletion | This work |
| EAEC 042 *irmA_4509*xFLAG *oxyR* | 042 *irmA_4509*xFLAG carrying *oxyR* deletion | This work |
| EAEC 042 *irmA_4509*xFLAG *irmA_2244* | 042 *irmA_4509*xFLAG carrying *irmA_2244* deletion | This work |
| EAEC 042 *irmA_4509*xFLAG *p4509* | 042 *irmA_4509*xFLAG carrying *irmA_4509* promoter deletion | This work |
| EAEC 042 *irmA_4509*xFLAG *p4511* | 042 *irmA_4509*xFLAG carrying *agn_4511* promoter deletion | This work |
| EAEC 042 Δ*LC* | 042 derivative carrying *lacZ and cat* deletion | This work |
| EAEC 042 Δ*LC* *irmA_2244::lacZ* | 042 Δ*LC* carrying the *lacZ* reporter gene within the *irmA_2244* coding sequence | This work |

| Bacterial strain | Description | Source/Reference |
|---|---|---|
| EAEC 042 Δ*LC* *irmA_2244*::*lacZ oxyR* | 042 Δ*LC irmA_2244*::*lacZ* derivative carrying *oxyR* deletion | This work |
| EAEC 042 Δ*LC* *irmA_2244*::*lacZ* *irmA_4509* | 042 Δ*LC irmA_2244*::*lacZ* derivative carrying *irmA_4509* deletion | This work |
| EAEC 042 Δ*LC* *irmA_2244*::*lacZ* *irmA_4509 oxyR* | 042 Δ*LC irmA_2244*::*lacZ* derivative carrying *irmA_4509* and *oxyR* deletions | This work |
| EAEC 042 Δ*LC* *irmA_4509*::*lacZ* | 042 Δ*LC* carrying the *lacZ* reporter gene within the *irmA_4509* coding sequence | This work |
| EAEC 042 Δ*LC* *irmA_4509*::*lacZ oxyR* | 042 Δ*LC irmA_4509::lacZ* derivative carrying *oxyR* deletion | This work |
| EAEC 042 Δ*LC* *irmA_4509*::*lacZ* *irmA_2244* | 042 Δ*LC irmA_4509::lacZ* derivative carrying *irmA_2244* deletion | This work |
| EAEC 042 Δ*LC* *irmA_4509*::*lacZ* *irmA_2244 oxyR* | 042 Δ*LC irmA_4509::lacZ* derivative carrying *irmA_2244* and *oxyR* deletions | This work |
| EAEC 042 *irmA_2244* | 042 derivative carrying *irmA_2244* gene deletion | This work |
| EAEC 042 *irmA_4509* | 042 derivative carrying *irmA_4509* gene deletion | This work |
| EAEC 042 *irmA_2244* *irmA_4509* | 042 derivative carrying *irmA_2244* and *irmA_4509* deletions | This work |
| EAEC 042 *oxyR* | 042 derivative carrying *oxyR* deletion | This work |
| EAEC 042 *irmA_2244* *oxyR* | 042 derivative carrying *irmA_2244* and *oxyR* deletions | This work |
| EAEC 042 *irmA_4509* *oxyR* | 042 derivative carrying *irmA_4509* and *oxyR* deletions | This work |

**Supplementary Table 1.** Bacterial strains used in this work.

| Plasmid | Description | Source/Reference |
|---|---|---|
| pKD3 | *bla* FRT *cat* FRT PS1 PS2 oriR6K Cm$^r$ Cb$^r$ | (3) |
| pKD4 | *bla* FRT *ahp* FRT PS1 PS2 oriR6K Km$^r$ Cb$^r$ | (3) |
| pKD46 | *bla* P$_{BAD}$ *gam bet exo* pSC101 oriTS Cb$^r$ | (3) |
| pCP20 | *bla cat cl857 lP*$_R$*flp* pSC101 oriTS Cb$^r$ | (4) |
| pSUB11 | R6KoriV -FLAG and -Kmr coding template vector | (5) |
| pLATE31 | P$_{T7}$ lacO *rep* (pMB1) *bla* (Cb$^r$) 6xHis C-terminal | aLICator LIC Cloning & Expression System (*ThermoScientific*) |
| pKG136 | *ahp km* FRT l*acZY+* t$_{his}$ oriR6K | (6) |

| Plasmid | Description | Source/Reference |
|---|---|---|
| pUJ8 | *trp'-'lacZ* promoterless vector | (7) |
| pUJ8-p2242 | pUJ8 with *agn43_2242* promoter region | This work |
| pUJ8-p4511 | pUJ8 with *agn43_4511* promoter region | This work |
| pUJ8-p2244 | pUJ8 with *irmA_2244* promoter region | This work |
| pUJ8-p4509 | pUJ8 with *irmA_4509* promoter region | This work |
| pUJ8-mut*p4509 | pUJ8 + *irmA_4509* promoter region with site-directed mutagenesis | This work |

**Supplementary Table 2.** Plasmids used in this work

| Oligonucleotide name | Sequence 5' → 3' | Use |
|---|---|---|
| *irmA* pLATE31CT fw | AGAAGGAGATATAACTATGATTCACCTGTTCAAAACCTGCATGATTACC | Cloning of *irmA* gene in pLATE31 vector |
| *irmA* pLATE31CT rv | GTGGTGGTGATGGTGATGGCCGTTAACGTTTTTTCCGGAAACGGTAAT | Cloning of *irmA* gene in pLATE31 vector |
| LIC forward sequencing primer | TAATACGACTCACTATAGGG | Confirmation of cloning in pLATE31 vector and sequencing |
| LIC reverse sequencing primer | GAGCGGATAACAATTTCACACAGG | Confirmation of cloning in pLATE31 vector and sequencing |
| 2244 P1 | GTGTCATCGTCATGACTCAGAGAGGTAAATACCATGATTCACGTGTAGGCTGGAGCTGCTTC | *irmA_2244* deletion |
| 4509 P1 | GTGTCATCGTCATGATTCAGAGAGGTAAATACCATGATTCACGTGTAGGCTGGAGCTGCTTC | *irmA_4509* deletion |
| *irmA* P2 | ACAGAGGCTGAAGCATGAAACTGACTTCAGGGATCAGTTAACCATATGAATATCCTCCTTAGT | *irmA_2244* or *irmA_4509* deletion |
| 2244 P1up | CCGGCGCAACGGATCTTCAAC | *irmA_2244* deletion confirmation |
| 2244 P2down | GTTTTTCAGGAACGAAAGCTG | *irmA_2244* deletion confirmation |
| 4509P1up | GACGGGGGAAAACATTTCAGT | *irmA_4509* deletion confirmation |
| 4509P2down | GTCATCATCAGAAATGGAACG | *irmA_4509* deletion confirmation |
| IrmA 3X P1 | CCATCCACTGCTAATTACCGTTTCCGGAAAAAACGTTAACGACTACAAAGACCATGACGG | *irmA_2244* or *irmA_4509* FLAG insertion |
| IrmA 3X P2 | ACAGAGGCTGAAGCATGAAACTGACTTCAGGGATCAGTTACATATGAATATCCTCCTTAG | *irmA_2244* or *irmA_4509* FLAG insertion |

| Oligonucleotide name | Sequence 5' → 3' | Use |
|---|---|---|
| *hns* P1 | ATGAGCGAAGCACTTAAAATTCTGAAC AACATCCGTACTCTTGTGTAGGCTGGA GCTGCTTC | *hns* deletion |
| *hns* P2 | TTATTGCTTGATCAGGAAATCGTCGAG GGATTTACCTTGCTCCATATGAATATCC TCCTTAGT | *hns* deletion |
| *hns* P1up | CCACCCCAATATAAGTTTGAG | *hns* deletion confirmation |
| *hns* P2down | GGGATTTTAAGCAAGTGCAATC | *hns* deletion confirmation |
| *hha* P1 | ATGTCCGAAAAACCTTTAACGAAAACC GATTATTTAATGCGTGTGTAGGCTGGA GCTGTCTTC | *hha* deletion |
| *hha* P2 | TTAGCGAATAAATTTCCATACTGAGGA AGGGATCTTGTCGTACATATGAATATC CTCCTTAGT | *hha* deletion |
| *hha* P1up | GTTAGAATTATTACAACCATGGG | *hha* deletion confirmation |
| *hha2* P1 | ATCACGTTAATTGCAGCATAAAGTGAT GAGAGGCTAATGGAAGTGTAGCTGGA GCTGCTTC | *hha2* deletion |
| *hha2* P2 | AACGCCCGGCACAATACCGCTGTGATT AACGAACGTTCAGGTCATATGAATATC CTCCTTAGT | *hha2* deletion |
| *hha2* P1up | GTCTGAGCGGTAAACGTATC | *hha2* deletion confirmation |
| KT | CGGCCACAGTCGATGAATCC | Km cassette insertion |
| *oxyR* P1 | GCCATGAACTATCGTGGCGATGGAGG ATGGATAATGAATATTGTGTAGGCTGG AGCTGCTTC | *oxyR* deletion |
| *oxyR* P2 | GCGGAAGCCTATCGGGTAGCTGCGCT AAATGGCTTAAACCGCCATATGAATAT CCTCCTTAGT | *oxyR* deletion |
| *oxyR* P1up | GATAGGGATAATCGTTCATTG | *oxyR* deletion confirmation |
| *oxyR* P2down | GCAATACTATTGAGTACTTCG | *oxyR* deletion confirmation |
| A3 adapter | GAUAUGGCGGAAUUCCUGUAGAACG AACACUAGAAGAAA | 5' RACE |
| B6 | GCGCGAATTCCTGTAGA | 5' RACE |
| *irmA* rv | GCGACATTTTCCACGCACTC | ***Walking RT-PCR*** |
| *yeeR* fw | GAAATATCAGTGCCCTGAAC | *Walking RT-PCR* |
| Int fw | TTGTCAGTGTCATCGTCATG | ***Walking RT-PCR*** |
| *irmA* fw | GCATGATTACCGCCTTCATT | *Walking RT-PCR* |

| Oligonucleotide name | Sequence 5' → 3' | Use |
|---|---|---|
| prom2242 fw *EcoRI* | CGGAATTCACCTGTCGTGACTGATGCCCT | Cloning of *agn43_2242* promoter in pUJ8 vector |
| prom2242 rv *BamHI* | CGGGATCCCAGCTTTTCCTTAGATTGAGG | Cloning of *agn43_2242* promoter in pUJ8 vector |
| prom4511 fw *EcoRI* | CGGAATTCACCTGTTGTGGATGATGTCCT | Cloning of *agn43_4511* promoter in pUJ8 vector |
| prom4511 rv *BamHI* | CGGGATCCTTCCTTAATCAGAGTGAGGGT | Cloning of *agn43_4511* promoter in pUJ8 vector |
| promirmA fw *EcoRI* | CGGAATTCAAGAAGCATTACCCCCGGAGT | Cloning of *irmA* promoter in pUJ8 vector |
| promirmA rv *BamHI* | CGGGATCCTCATGACGATGACACTGACAA | Cloning of *irmA* promoter in pUJ8 vector |
| pUJ8 p1up | CTGTAAGCGGATGCCGGGAGCA | Cloning confirmation and sequencing |
| lacZR | GATGACCTGCAAGGCGATTA | Cloning confirmation and sequencing |
| promirmA P1 | ATATGGTTAACCGGCTGGAAGAAGCATTACCCCCGGAGTTACGTGTAGGCTGGAGCTGCTTC | *irmA* promoter deletion |
| promirmA P2 | TTACCTCTCTGAATCATGACGATGACACTGACAAGTCAGGTGCATATGAATATCCTCCTTAGT | *irmA* promoter deletion |
| promirmA P1up | CACTGGTGGTCAGTATCATC | *irmA* promoter deletion confirmation |
| prom4511 P1 | GTTGCCCGCGCGGTATGGAACTGGATCTTCTTCTGAACCTGTGTGTAGGCTGGAGCTGCTTC | *agn43_4511* promoter deletion |
| prom4511 P2 | CCTGTAGCAGGTATTCAGATGTCGTTTCATCAGCATTTCCTTCATATGAATATCCTCCTTAGT | *agn43_4511* promoter deletion |
| prom4511 P1up | GTGGACCGGATATTTGACAC | *agn43_4511* promoter deletion confirmation |
| prom4511 P2down | TGCCCGCCATGTTTAATGGT | *agn43_4511* promoter deletion confirmation |
| Mut_prom_4509 fw | ACCGGCATAACGGCTATTCTGCGCCTTTTACGCTTTATCACCTGA | Site-directed mutagenesis |
| Mut_prom_4509 rv | TCAGGTGATAAAGCGTAAAAGGCGCAGAATAGCCGTTATGCCGGT | Site-directed mutagenesis |

**Supplementary Table 3.** Oligonucleotides used in this work.

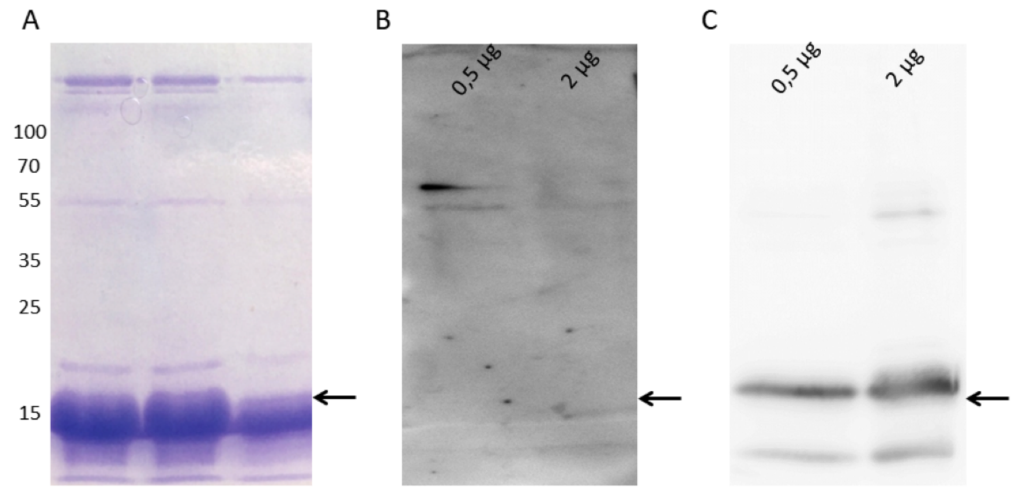


**Supplementary Figure 1.** Purification of the IrmA protein. A) Coomassie blue staining of three eluates performed with A200 buffer. B) Immunodetection of purified IrmA protein using unimmunized rabbit serum. Two concentrations of the IrmA protein are indicated. C) Immunodetection of the purified IrmA protein using rabbit serum immunized with the IrmA protein. Two different concentrations of the purified IrmA protein are shown. The arrow indicates the position of the IrmA protein.

```
flu_2242  ACCTGTCGTGACTGATGCCCTCCCTGACTCTGAGTCTGCTCACAAAAGCACTGTTTTCGT
flu_4511  ACCTGTTGTGGATGATGTCCTCCCTGCCTCTGAGTCTGCTCACAAAAGCGCTGTTTTCGT
          ****** ***  ***** ******** ********************** **********

flu_2242  TACTGTCTCTCTTGTCCGTGCAATAGCTCAATAATAGAATAAAACGATCGATATCTATTT
flu_4511  TACTGTCTCTCTTGTCCGTGCAATAGCCTGATAATAGAATAAAACGATCGATACCTATTT
          ***************************   *********************** ******

flu_2242  TATCGATCGTTTATATCGATCGATAAGCTAATAATAACCTTTGTCAGTAACATGCACAGA
flu_4511  TATCGATCGTTTATATCGATCGTCATGCTAATAATAACTCCTGTTAGCAACGTGCGCAGA
          **********************  * ************   *** ** *** *** ****

flu_2242  TACGTACAGAAAGAC-ATTCAGGGAACAACAGAACCACAATTCAG-AAACTCCCACAGCC
flu_4511  TACACACAGACATGAGATTCAGGGAACAACAGAGCCACACGTCAGAAACTTCCGTCAGCC
          ***  ***** *    ***************** *****  **** **  ***  *****

flu_2242  GGACCTCCGGCACTGTAACCCTTTACCTGCCGGTATCCACGTTTGTGGGTACCGGCTTTT
flu_4511  GGACCTCCGGCACTGTAACCCTTTACCTGCCGGTATCCACATCTGTGGATACCGGCTTTT
          **************************************** * ***** ***********

flu_2242  TTATTCACCCTCAATCTAAGGAAAAGCTG
flu_4511  TTATTCACCCTCACTCTGATTAAGGAA--

              ************* *** *  **
                                   :
                                   :
                                   :

irmA_2244  CTGGTGGTCAGTATCATCAGGCAACGCCCCGTCATGGAATATGATGCATCCCATCTCGGC
irmA_4509  CTGGTGGTCAGTATCATCAGGCAACGCCCCGTCATGGAATATGATGCATCCCATCTCGGC
           ************************************************************

irmA_2244  AATATGGTTAACCGACTGGAAGAAGCATTACCCCCGGAGTTACCGGCATAACGGCTATTC
irmA_4509  AATATGGTTAACCGGCTGGAAGAAGCATTACCCCCGGAGTTACCGGCATAACGAGTATTC
           ************** **************************************  *****

irmA_2244  TGCGCCTTTTACGCTTTATCACCTGATTTGTCAGTGTCATCGTCATGACTCAGAGAGGTA
irmA_4509  TGCACCTTTTACGTTTTATCACCTGACTTGTCAGTGTCATCGTCATGATTCAGAGAGGTA
           *** ********* ************ ********************** ***********

irmA_2244  AATACCATGATTCACCTGTTCAAAACCTGCATGATTACCGCCTTCATTCTGGGGTTAACG
irmA_4509  AATACCATGATTCACCTGTTCAAAACCTGCATGATTACCGCCTTCATTCTGGGGTTAACG
           ************************************************************

irmA_2244  TGGTCTGCCCCACTCCGGGCACAGGATCAACGTTACATCAGTATACGCAATACAGATACG
irmA_4509  TGGTCTGCCCCACTCCGGGCACAGGATCAACGTTACATCAGTATACGCAATACAGATACG
           ************************************************************
```

**Supplementary Figure 2.** Nucleotide differences in the regulatory regions of the *flu* and *irmA* alleles. OxyR and Dam (GATC) binding sites are shown in bold and underlined. The -10 and -35 boxes corresponding to the previously described *flu* promoter are labeled in yellow. The *yeeR* stop codon is labeled in purple, and the *irmA* start codon is labeled in green.

```
2244   CTGGTGGTCAGTATCATCAGGCAACGCCCCGTCATGGAATATGATGCATCCCATCTCGGC
4509   CTGGTGGTCAGTATCATCAGGCAACGCCCCGTCATGGAATATGATGCATCCCATCTCGGC
       ************************************************************

2244   AATATGGTTAACCGACTGGAAGAAGCATTACCCCCGGAGTTACCGGCATAACGGCTATTC
4509   AATATGGTTAACCGGCTGGAAGAAGCATTACCCCCGGAGTTACCGGCATAACGAGTATTC
       ************** **************************************  *****

2244   TGCGCCTTTTACGCTTTATCACCTGATTTGTCAGTGTCATCGTCATGACTCAGAGAGGTA
4509   TGCACCTTTTACGTTTTATCACCTGACTTGTCAGTGTCATCGTCATGATTCAGAGAGGTA
       *** ********* ************ ********************** **********

2244   AATACCATGATTCACCTGTTCAAAACCTGCATGATTACCGCCTTCATTCTGGGGTTAACG
4509   AATACCATGATTCACCTGTTCAAAACCTGCATGATTACCGCCTTCATTCTGGGGTTAACG
       ************************************************************

2244   TGGTCTGCCCCACTCCGGGCACAGGATCAACGTTACATCAGTATACGCAATACAGATACG
4509   TGGTCTGCCCCACTCCGGGCACAGGATCAACGTTACATCAGTATACGCAATACAGATACG
       ************************************************************
```

**Supplementary Figure 3**. Identification of the *irmA* promoter by using BPROM software. The -10 and -35 boxes are highlighted in yellow. The *yeeR* stop codon is labeled in purple, and the *irmA* start codon is labeled in green.

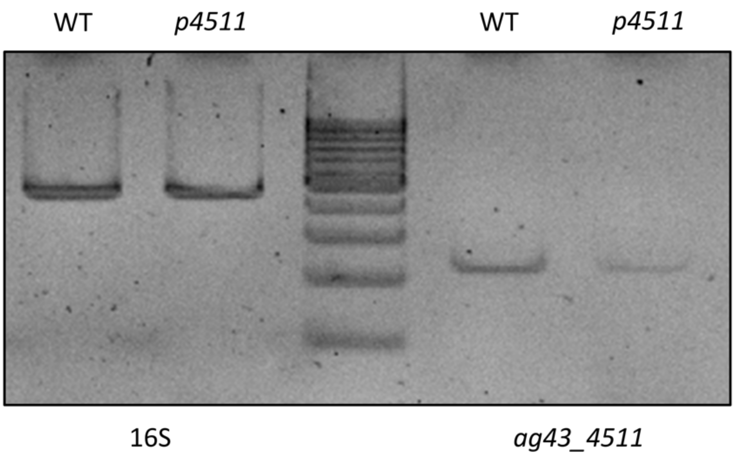


**Supplementary Figure 4.** Deletion of the *flu* promoter results in a reduced transcription of the *flu* 4511 allele. A 315 bp sequence including the *flu* promoter of the *flu* 4511 allele was deleted in the wt 042 strain. Transcription of the *flu* 4511 allele both in the wt 042 strain and in its derivative lacking the *flu* promoter was determined by isolating RNA, followed by its

retrotranscription to cDNA. Thereafter, transcripts corresponding to both the 16S RNA and the *flu* 4511 allele were amplified with specific primers. Bands correspond to the amplification of the corresponding transcripts in the wild-type 042 strain (WT) and in its mutant derivative lacking the *flu* promoter (*p4511*).

**References**


1. Taylor RG, Walker DC, Mclnnes RR. 1993. *E.coli* host strains. Nucleic Acids Res 21:1677–1678.
2. Studier FW, Moffatt BA. 1986. Use of bacteriophage T7 RNA polymerase to direct selective high-level expression of cloned genes. J Mol Biol 189:113–130.
3. Datsenko KA, Wanner BL. 2000. One-step inactivation of chromosomal genes in *Escherichia coli* K-12 using PCR products. Proc Natl Acad Sci U S A 97:6640–6645.
4. Cherepanov PP, Wackernagel W. 1995. Gene disruption in *Escherichia coli*: TcR and KmR cassettes with the option of Flp-catalyzed excision of the antibiotic-resistance determinant. Gene 158:9–14.
5. Uzzau S, Figueroa-Bossi N, Rubino S, Bossi L. 2001. Epitope tagging of chromosomal genes in *Salmonella*. Proc Natl Acad Sci U S A 98:15264–15269.
6. Ellermeier CD, Janakiraman A, Slauch JM. 2002. Construction of targeted single copy lac fusions using λ Red and FLP-mediated site-specific recombination in bacteria. Gene 290:153–161.
7. De Lorenzo V, Herrero M, Jakubzik U, Timmis KN. 1990. Mini-Tn5 transposoon derivatives for insertion mutagenesis, promoter probing, and chromosomal insertion of cloned DNA in gram-negative eubacteria. J Bacteriol 172:6568–6572.
